# Leprosy among schoolchildren in the Amazon region: A cross-sectional study of active search and possible source of infection by contact tracing

**DOI:** 10.1371/journal.pntd.0006261

**Published:** 2018-02-26

**Authors:** Valderiza Lourenço Pedrosa, Luiz Claudio Dias, Enrique Galban, André Leturiondo, Jamile Palheta, Monica Santos, Milton Ozório Moraes, Carolina Talhari

**Affiliations:** 1 Departamento de Ensino e Pesquisa, Fundação Alfredo da Matta, Manaus, Brazil; 2 Departamento de Epidemiologia, Facultad de Medicina Calixto García, La Habana, Cuba; 3 Escola de Ciências da Saúde, Universidade do Estado do Amazonas, Manaus, Brazil; 4 Laboratório de Hanseníase, Fundação Oswaldo Cruz, Rio de Janeiro, Brazil; Swiss Tropical and Public Health Institute, SWITZERLAND

## Abstract

**Background:**

The high rate of leprosy cases among children under 15 years of age in Brazil indicates ongoing transmission within the community. The identification of the new leprosy cases among contacts can help identify the source of infection and interrupt the transmission chain. This study aims to determine the detection rate of previously undiagnosed cases of leprosy among schoolchildren who are under 15 years of age living in Manaus, Amazonas, Brazil, and their possible source of infection by contact tracing.

**Methodology/Principal findings:**

This was a school-based, cross-sectional study in which the identification of active leprosy cases was conducted in 277 out of 622 randomly selected public schools in Manaus, Amazonas, Brazil. Suspected cases of leprosy were referred to the Alfredo da Matta Foundation, a reference center for leprosy in Manaus. A total of 34,547 schoolchildren were examined, and 40 new leprosy cases were diagnosed. Among new cases, 57.5% were males, and 80.0% demonstrated paucibacillary leprosy. A total of 196 of 206 registered contacts were screened, and 52.5% of the newly diagnosed children’s cases had at least one positive household contact. In these contacts, grandparents (52.4%) were the most common co-prevalent cases, while 14.3% were uncles, 9.5% were parents and 9.5% were granduncles. Seven contacts (5.0%), including four siblings of child patients were newly diagnosed. Our data indicate that the prevalence is 11.58 per 10,000, which is 17 times higher than the registered rate.

**Conclusions/Significance:**

This study suggests that the detection rate of leprosy among schoolchildren may have remained unchanged over the past thirty years. It also indicates that that active case finding is necessary for reaching the World Health Organization’s goals of zero detection among children, especially in endemic areas where the prevalence of leprosy is obscure. Moreover, we assert that all children must have their household contacts examined in order to identify the possible source of infection and interrupt the disease’s transmission. Novel strategies to reinforce contact tracing associated with large-scale strategies of chemo- and immune-prophylaxis should be expanded to prevent the perpetuation of the disease cycle.

## Author summary

Leprosy is a disease that has long since been eradicated in the developed world, but it still affects poor people in developing countries, such as India, Brazil, and Indonesia. Because the causative agent of the disease may involve the skin and peripheral nerves, the disease can cause physical disabilities and deformities. Although leprosy affects all ages, children under 15 years of age are an important epidemiological marker because infection in that age group indicates active transmission within the community. In our work, we examined 34,547 children from public schools in Manaus, a city in the north of Brazil. In this population, we found 40 new cases of leprosy that were further confirmed by clinical and laboratorial tests. We also examined 196 people who had familiar or close non-familiar contact with the affected children. Among them, we identified the possible source of infection of 21 affected children and found seven new cases of leprosy. Overall, our findings revealed a detection rate of leprosy cases that was 17 times higher than the registered number. This indicates the necessity of identifying active cases of leprosy in order to improve case detection and effectively control the disease.

## Introduction

*Mycobacterium leprae*, the causative agent of leprosy, is primarily transmitted person-to-person and through the air. People living in leprosy-endemic regions are at greater risk of being exposed to the infection. The risk of developing the disease among paucibacillary (PB) contacts is 2–3 times higher than that of the general population, while the risk increases to 5–10 times among multibacillary (MB) contacts[1–3]. Therefore, contact tracing not only results in the detection of additional cases but further offers several indirect advantages such as early diagnosis and reduced risk of transmission[4].

Familial leprosy distribution indicates a relationship between the clinical forms of the disease and kinship degree. Consanguineous relatives belonging to families whose fathers or mothers had lepromatous leprosy showed a higher risk of developing the same type of disease. On the other hand, non-consanguineous relatives were at a higher risk of contracting other clinical forms of the disease [5]. A study conducted in the Philippines showed that the risk of developing lepromatous leprosy was three times higher when one of the parents presented with this clinical form of the disease [6]. In leprosy hyperendemic areas, the risk of developing the disease may be elevated not only for household contacts but also for the residents in neighboring homes [7, 8]. Recently, a survey conducted in a hyperendemic Brazilian region demonstrated no significant difference in detection rates between household contacts and neighbors [8].

According to the World Health Organization (WHO), Brazil accounts for more than 80% of leprosy cases diagnosed in the Americas[9]. In 2016, 25,218 new leprosy cases were diagnosed in Brazil, and 1,696 (6.7%) of those individuals were children, which corresponds to a diagnosis rate of 3.63 per 10,000 people. In the same year, Amazonas State reported 443 new cases of leprosy; the diagnosis rate was 1.10/10,000 inhabitants, and this was considered highly endemic by the Brazilian Ministry of Health (BMH)[10]. Although the introduction of multidrug therapy (MDT) in the beginning of the 1980s drastically influenced the total number of cases, there has been stagnation and a slight decrease in incidence over the past 10 years. This data suggests that it is likely that MDT has little impact on incidence because transmission occurs prior to diagnosis. Thus, strategies to prevent leprosy transmission indicate that contact tracing and post-exposure prophylactic protocols using rifampicin and/or BCG[11–13] should be successful. In this regard, the diagnosis of leprosy cases among children under 15 years of age can help provide estimates of ongoing transmission [14, 15] and the presence of active disease foci in the community[16]. In the early 1980s, an active case finding in Manaus indicated a detection rate of 10.6 cases of leprosy per 10,000 children [17].

This study was carried out to identify previously undiagnosed cases of leprosy among schoolchildren and their possible source of infection by contact tracing. Patterns of family contact with leprosy are demonstrated through genograms.

## Methods

### Population and study design

This was a school-based, cross-sectional study. Active case finding of leprosy in children under 15 years of age was conducted from March 2014 to December 2016 in 277 of the 626 public schools in Manaus, Amazonas, Brazil. Manaus is one of the major cities in the north of Brazil and has approximately 2,800,000 inhabitants [18]. The metropolitan area of Manaus has 626 public schools that enroll approximately 250,000 children [19]. Target schools were randomly chosen through a lottery method using Open Source Epidemiologic Statistics for Public Health software to obtain the study population. The probabilistic sample of 30,352 students was calculated based on the target population of students; the sampling error was 0.03%, and the confidence interval was 95%.

### Eligible participants and recruitment process

Children from randomly selected public schools in Manaus, Amazonas, were eligible to participate in the study. The recruitment process started with an open seminar on leprosy and the purpose of the study. After written informed consent was obtained from parents or legal guardians, children received an initial physical examination conducted by trained and experienced leprosy and skin nursing technicians. The initial physical examination took place at school. Suspected cases of leprosy and other skin diseases, along with their legal guardians, were referred to the Alfredo da Matta Foundation (AMF), a referral center for leprosy and other skin diseases in Manaus.

Three dermatologists and laboratory tests confirmed the diagnosis of leprosy, which was initially based on the presence of leprosy’s cardinal signs, i.e., if the patient had one or more lesions with a definite loss of sensation and/or peripheral nerve thickening. If these signs were evident, diagnosis was confirmed by histopathological changes and analysis of bacillary loads in a slit skin smear test (SSS). Classification was performed according to Ridley and Jopling [20, 21]. In cases in which there was no confirmation through the previously mentioned routine tests, a polymerase chain reaction (PCR) was performed to detect *M*. *leprae* DNA, as previously described [22]. This technique has been used in patients who have clinical signs of leprosy but no confirmation through routine tests and histopathology, in difficult-to-diagnose cases, and in early detection in household contacts [22].

For confirmed cases of leprosy, a standardized questionnaire was administered to gather past medical history and social and demographic information, such as BCG scar status, data on household and dwelling contacts, race—white, black, yellow, brown or indigenous -, etc.) was applied. For treatment purposes, leprosy cases were classified as PB or MB, as recommended by the Brazilian Ministry of Health (BMZ) [14] and the WHO [23].

Household contacts were defined as a group of people who lives or have lived with a leprosy patient within the past five years. Direct and next-door neighbors, when indicated by legal guardians, were also considered contacts. All contacts were initially examined by the nursing technicians for clinical evidence of leprosy; the diagnosis of leprosy was also confirmed by three dermatologists and the aforementioned laboratory tests.

### Statistical analysis

Data were analyzed using Epi Info 7 software. Initial descriptive studies were performed through frequency tables, position measurements and variability. Pearson's chi-square test or Fisher's exact tests were used to analyze the categorical variables. The significance level was 0.05, and the confidence interval was 95%. The GenoPro version 3.0.0.7 software was used to create genograms in order to identify the probable source of infection of new leprosy cases.

### Ethical statement

Ethical approval was granted by the AMF Research and Ethics Committee. Written informed consent was obtained from parents or guardians of children enrolled in the study. Parents or guardians disclosed the diagnosis of leprosy to the respective contacts.

## Results

### School epidemiological survey

This study was conducted in 277 randomly selected public schools located in various districts of Manaus. In total, 34,547 children under 15 years of age were enrolled in the study. Overall, 18,770 (54.3%) were females and 15,777 (45.7%) were males. The mean age was 9.6 years (standard deviation [SD] = 2.58). Regarding the distribution by self-reported ethnicity, the majority (90.0%) of schoolchildren examined were brown with similar results obtained for both sexes. According to the Brazilian Institute of Geography and Census, 69% of the Amazonas State’s population is brown [19].The analysis for different proportions between sex and ethnicity did not show a statistical significance between browns and whites (*p* = 0.16).

Overall, 8.2% of the 34,547 schoolchildren examined had skin diseases. The most common skin disease was fungal (*n* = 955; 33.8%), followed by eczema/dermatitis (*n* = 725, 25.6%), viral diseases (*n* = 153, 5.4%), and leprosy (*n* = 40, 1.4%). A total of 40 leprosy cases were identified out of the total number of schoolchildren that were examined, resulting in a prevalence of 11.58 per 10,000 people. Among them, 23 (57.5%) patients were males, and 17 (42.5%) were females. Regarding leprosy classification, 32 (80%) and 8 (20%) patients had PB and MB forms of leprosy, respectively; among the PB patients, 24 (60%) presented with one lesion. The analysis for different proportions between sex and leprosy classification did not show a statistical significance between PB and MB leprosy (*p* = 0.43). The mean age was 10.6 years (range 4–13) (Table 1).

**Table 1:** Clinical and epidemiological aspects of the 40 newly diagnosed cases of leprosy among schoolchildren in Manaus, Amazonas, Brazil.

Characteristics	Paucibacillary		Multibacillary		Total		p
	n = 32	%	n = 8	%	n = 40	%	
Sex							
Male	17	53,1	6	75,0	23	57,5	
Female	15	46,9	2	25,0	17	42,5	
Age Range							
5–7	1	3,1	2	25,0	3	7,5	
8–10	9	28,1	5	62,5	14	35,0	
11–14	22	68,8	1	12,5	23	57,5	0,004
Race/Color							
Brown	28	87,5	8	100,0	36	90,0	
White	4	12,5	0	0,0	4	10,0	0,38
Number of Lesions							
1	24	75,0	0	0,0	24	60,0	
2–5	8	25,0	0	0,0	8	20,0	
> 5	0	0,0	8	100,0	8	20,0	
Disability grade							
Grade 0	32	100,0	6	75,0	38	95,0	
Grade I	0	0,0	1	12,5	2	5,0	
Grade II	0	0,0	1	12,5	0	0,0	

This table displays sex, age, race, number of lesions and disability grade of the 40 newly diagnosed cases of leprosy.

Among the total cases of leprosy, 23 (59.0%) were 11 to 14 years of age; a similar pattern was found in both genders. According to data from the Municipal and State Education Departments of the State of Amazonas, 334,228 (68%) of the students are within this age range [19]. The analysis of the distribution among these three age groups showed statistically significant differences (*p* < 0.01). Notably, the majority of MB leprosy cases (*n* = 7) were less than 11 years of age. There was a predominance of the brown race among the cases (90.0%), and a similar result was obtained for both genders.

### Laboratory confirmation of clinically diagnosed cases

**Fig 1:**
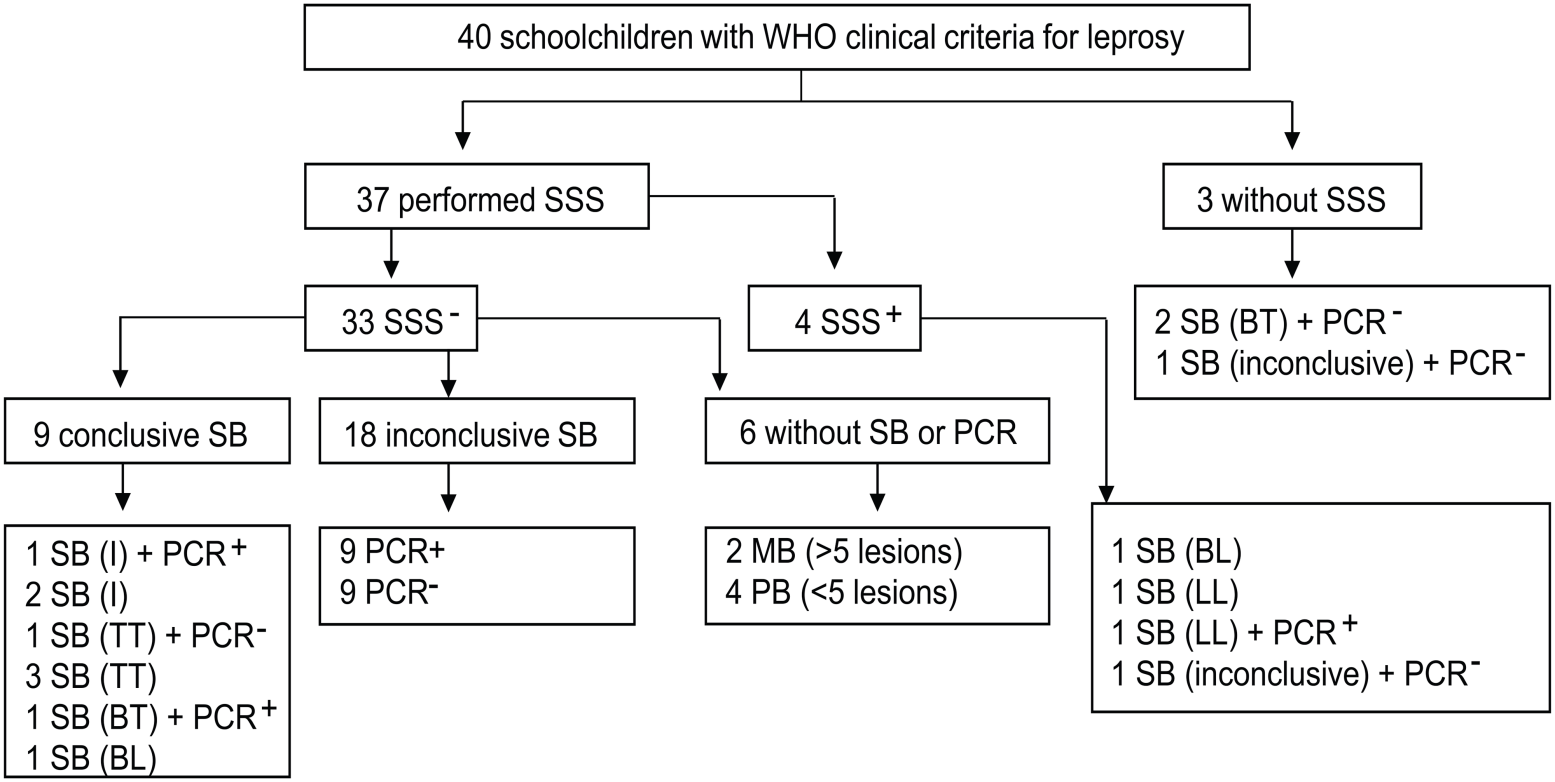
Scheme of laboratory tests performed to support clinical diagnosis. Of the 40 newly diagnosed cases of leprosy, 37 patients received a SSS test, 34 had histopathological examinations, and 26 had a PCR. SB: skin biopsy; SSS: slit skin smear; I: indeterminate; TT: tuberculoid-tuberculoid; BT: borderline-tuberculoid; BL: borderline-lepromatous; LL: lepromatous-lepromatous; MB: multibacillary; and PB: paucibacillary leprosy.

An overview of the tests performed to support diagnosis is presented in Fig 1. Of the 40 leprosy cases, 32 (80.0%) were PB, and 8 (20.0%) were MB. A SSS was performed in 37 (92.5%) of the leprosy cases, histopathological examination was performed in 34 (84.6%) cases, and PCR was performed in 26 (65.0%) cases. Six had a negative SSS test, but did not have either a skin biopsy nor PCR examination. Because these patients had skin lesions that fulfilled WHO clinical criteria for leprosy (four had up to five lesions and two had more than five lesions), they were diagnosed and treated for PB and MB leprosy, respectively.

Of the 37 patients who received a SSS, 33 (89.2%) were negative, and four (10.8%) were positive. Three patients did not undergo SSS, but these patients had a skin biopsy taken and presented a negative result for PCR. Histopathological features of leprosy were seen in two cases, and the result was inconclusive in one case. Because the latter patient had one lesion in which leprosy was clinically confirmed, he was given PB treatment.

Leprosy was confirmed by histopathological examination in 14 children from the 34 histopathological slides analyzed. The results were as follows: indeterminate (three cases), tuberculoid-tuberculoid (four cases), borderline-tuberculoid (three cases), borderline-lepromatous (two cases) and lepromatous-lepromatous (two cases). In 20 patients, the histopathological examination yielded results that were inconclusive but did not exclude the diagnosis of leprosy. In this group, nine patients had a positive PCR result, as they exhibited fewer than five lesions that were being treated for PB leprosy, and 11 patients were negative for PCR examination. One out of the 11 had a positive skin smear, and 10 were clinically diagnosed as leprosy cases. The patient with the positive skin smear was treated for MB leprosy, and the other 10 who presented with fewer than three lesions, received PB treatment. In total, *M*. *leprae* DNA was detected in 12 (46.2%) out of 26 patients.

BCG scar status was recorded as positive in the majority (*n* = 37, 92.5%) of the patients. Thirty-eight (95.0%) children demonstrated a zero incapacity grade; one case of paresthesia (grade one disability) and one case of ulnar claw (grade two disability) were detected. Both patients had MB leprosy.

### Contact tracing and diagnosis

A total of 206 people were registered as household contacts of the schoolchildren diagnosed with leprosy; only two direct neighbors, both with a past medical history of MB leprosy, were indicated as contacts by the legal guardians. Overall, 196 (95.1%) of the contacts were clinically examined. Among these contacts, we diagnosed seven new leprosy cases: five siblings, an uncle, and an aunt were detected as index case patients. Two of these contacts were also under 15 years of age. Three contacts had PB leprosy, and four, including the two children who were under 15 years of age, presented with MB leprosy.

Regarding the households, 21 (52.5%) patients lived with three to four people, whereas nine (22.5%) lived with more than seven people. More than 50% of the children lived with their families in households with up to four rooms, whereas 10 (25.0%) children lived in households with five or more rooms.

Among 40 schoolchildren diagnosed with leprosy, we were able to identify 21 (52.5%) who had or continued to have contact with patients within their household, familiar or not, who had previously been treated for leprosy or were still under leprosy treatment. Of these, six (28.6%) children had contact with grandparents with a past medical history of leprosy. Three (14.3%) had contact with uncles; two (9.5%) had contact with parents; two (9.5%) had contact with their granduncles; one (4.8%) had contact with an aunt; one (4.8%) had contact with a great-grandfather; one (4.8%) had contact with a grandmother and two cousins; and one (4.8%) had close contact with a neighbor who was receiving leprosy treatment. Notably, the father of four sibling schoolchildren (19.0%) was receiving leprosy treatment, while a grandmother and a great-grandfather had already been treated for leprosy. All of them, including the recently diagnosed siblings, presented with MB leprosy. Nevertheless, we understand that we did not design the study to test the familial/genetic nature of the susceptibility. However, we were able to observe important clusters where the physical distance and familial distance were detected. This description reinforces the need for contact tracing to stop leprosy transmission. As for the other 19 schoolchildren diagnosed with leprosy during this survey, we were not able to identify the possible source of infection.

**Fig 2:**
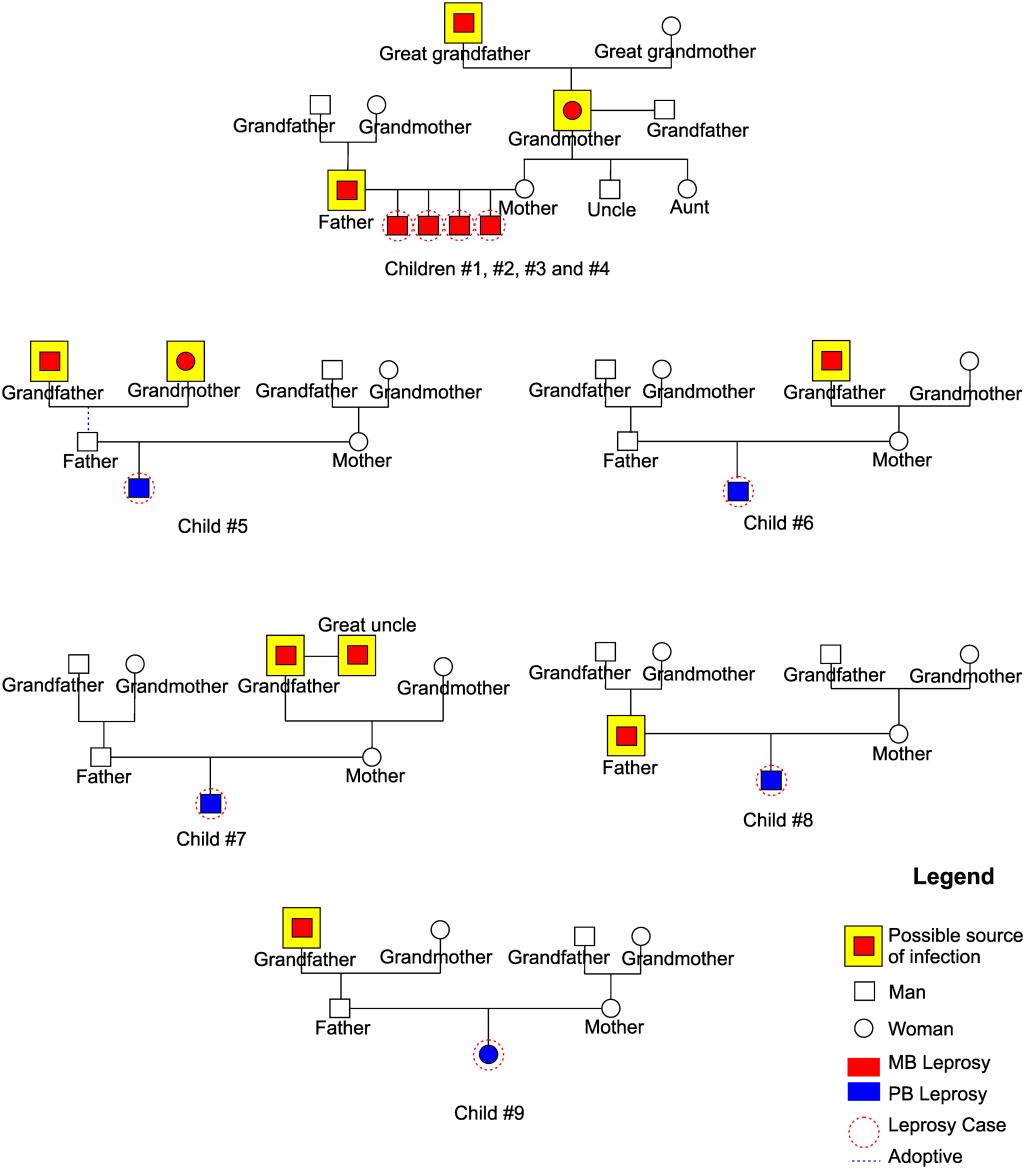
Genograms of schoolchildren #1, #2, #3, #4, #5, #6, #7, #8 and #9. Genograms showing the possible source of infection with *M*. *leprae* among nine newly diagnosed leprosy cases.

Fig 2 shows genograms of nine out of 40 schoolchildren with leprosy and their possible sources of infection. Children #1, #2, #3 and #4 belonged to the same family; during the investigation, it was found that the father, the maternal grandmother and the great-grandfather had been treated for leprosy, and the first two relatives were MB. Children #5, #6, #7, and #21 had a history of grandfathers treated for MB leprosy (Fig 2). The parents of children #8 and #20 were receiving treatment for MB and PB leprosy, respectively (Fig 2).

## Discussion

This school-based, cross-sectional study found a higher leprosy prevalence among children than that registered in the official data. This result suggests that contact tracing is an important epidemiological tool in diagnosing new cases of the disease and possible sources of leprosy infection. In 2013, one year before we began enrolling children in our study, the prevalence of leprosy in this population in Manaus was 0.68 cases per 10,000 children (0.68/100,000 in Amazonas State and 0.50/100,000 in Brazil)[10,24]. Our study cannot estimate prevalence exactly. However, our data clearly indicate a hidden prevalence, since our data suggests that 11.58 per 10,000, which would be 17 times higher than that in the registered data. We detected 40 new cases of leprosy out of a total of 34,547 examined schoolchildren. New cases of leprosy diagnosed among screened contacts under 15 years of age were not included in the aforementioned prevalence. Overall, this data suggests the existence, in the city of Manaus, of a hidden prevalence of significant magnitude.

From 1979 to 1982, Talhari and co-authors performed an active case finding in Manaus and found a prevalence of 10.6 cases of leprosy per 10,000 children [17]. From 1991 to 2016, the birth rate decreased in Amazonas State, from 32.4 to 19.7 per 1,000, respectively [25, 26]. Accordingly, official data from BMH show that the leprosy prevalence among children has been declining for the past 25 years in Amazonas State and also in Brazil [10]. However, our data, if confirmed in a design to estimate the prevalence, it would likely to be even higher than that found over 30 years ago.

Recently, high rates of clinical[27] and subclinical leprosy have been reported in Brazil [28]. In both studies, the diagnosis of leprosy was based on clinical and serological results. In our study, the vast majority (85.0%) of new cases of leprosy were confirmed by at least one diagnostic method that combines classical and novel tools: SSS and/or skin biopsy and/or PCR test. Accurate diagnosis and careful description of previously undiagnosed leprosy cases are important to address the true prevalence of the disease in endemic countries.

In this study, 54.3% of screened schoolchildren were female, but the leprosy cases were male (57.5%); this data is in accordance to the official data from the BMZ [19, 24]. The majority of the leprosy cases were diagnosed in older children. This reinforces the need for active case finding and suggests that instituting an approach to contact tracing is probably a valuable policy.

The majority (80.0%) of the newly diagnosed cases of leprosy were PB with the presentation of a single lesion; this is similar to the rate found in other studies [27, 29, 30]. However, eight schoolchildren in addition to four contacts who were less than 15 years of age demonstrated MB leprosy. In endemic areas, the early exposure to *M*. *leprae* and the presence of familial cases of the disease facilitate the greater frequency of contamination of children [17, 31–33].

Of the 21 schoolchildren with leprosy whose possible source of infection was identified among household contacts, 95.2% had contact with family members who previously had or were still receiving treatment for the disease. Contact with infected grandparents was found to be the most probable source of infection in our study. Notably, we found a cluster in which three generations had been diagnosed and treated for leprosy. High rates of consanguinity were found in other studies [30, 34], wherein parents and grandparents were the most likely source of infection [35–37]. Although household contact with an MB case is the strongest known determinant of leprosy risk, the vast majority of such contacts never manifest disease, which indicates the crucial role of genetic and/or environmental factors in the transmission of the *M*. *leprae* infection and/or the pathogenesis of clinical leprosy[31].

It is worth highlighting that patients and families are frequently not aware of any contact they have had with the disease, and they are often unaware of leprosy patients in the family or in the nearby neighborhood. Patients with active disease and higher bacillary loads are considered the most important actors in transmitting and perpetuating the disease in a way that household contacts exhibit the highest risk of developing the disease. Therefore, screening family and non-family members in leprosy-affected households is mandatory. Also, chemo or immuno-prophylaxis has been shown to reduce the risk among the household contact population [11– 13].

In addition to current leprosy cases in the family, housing in endemic areas, agglomerations of people living in a single household, family and social aggregation habits, household features, unfavorable conditions in the population and low educational level [35] are known risk factors for leprosy. In our study, conducted in an endemic area, more than 70% of the families lived in households with up to four rooms, and approximately 18 (45%) of the cases cohabited with more than five people.

Although controversial [13, 38], the administration of an additional dose of BCG to all healthy contacts is still recommended [11, 14]. Besides reducing clinical leprosy among vaccinees, mainly of the MB type, recent data suggested that BCG vaccination of household contacts of MB leprosy patients may induce activation of T cell clones that recognize *M*. *leprae* specific antigens not shared with BCG [39]. The majority (92.5%) of schoolchildren diagnosed with leprosy in this study had one positive BCG scar and the PB form of the disease (80.0%). Perhaps, if these children had been examined and vaccinated when their relatives were diagnosed with leprosy, we would not have had them as patients. Furthermore, early diagnosis could have prevented the occurrence of disability as found in two of the newly diagnosed cases of leprosy in this study.

The frequency of leprosy occurrence in children is an important epidemiological indicator in determining the level of transmission of the disease. Recently, the WHO has published goals for the year 2020 suggesting that, among health control issues, leprosy should have zero cases in children and zero cases with incapacities [40]. Officially, a trend towards the decrease of leprosy among children under 15 years of age has been suggested. However, our data indicates that the true prevalence of leprosy in this particular population may be slightly higher than that found over 30 years ago in the city of Manaus. To stop transmission, programs for the screening of household contacts should be improved and expanded, as screening has proven to be efficient for detecting early cases of leprosy [4]. These approaches associated with BCG vaccination and/or single dose rifampicin (SDR) reduce new cases in this household contact group [11, 41]. However, complementary approaches to improve surveillance and, thus, uncover hidden undiagnosed infectious cases that are actively transmitting leprosy are crucial to break the chain of transmission. Here, we provide evidence that screening of schoolchildren could be a valuable strategy to support leprosy control and achieve the goal of zero transmission.

This study shows that living with or in close proximity to leprosy patients and large family agglomerations in households with few rooms may be important risk factors for leprosy transmission among children. Moreover, this study highlights the value of contact screening of leprosy patients. There is a high level of family contact with leprosy in these cases, which gives support to the strategy of screening children in leprosy-affected households.

## Supporting information

S1 Checklist STROBE checklist. (DOCX)

## References

[1] Talhari S, Penna GO, Gonçalves HS, Oliveira MLW. Hanseníase. 5. ed Manaus: Dilivros; 2015 pp. 1–6.

[2] Sundar Rao PS, Jesudasan K, Mani K, Christian M. Impact of MDT on incidence rates of leprosy among household contacts. Part 1. Baseline data. Int J Lepr Other Mycobact Dis. 1989;57: 647–51. 2778370

[3] de Matos HJ, Duppre N, Alvim MFS, Vieira LMM, Sarno EN, Struchiner CJ. Epidemiologia da hanseníase em coorte de contatos intradomiciliares no Rio de Janeiro (1987–1991). Cad Saude Publica. Escola Nacional de Saúde Pública, Fundação Oswaldo Cruz; 1999;15: 533–542. doi: 10.1590/S0102-311X1999000300010 10.1590/s0102-311x1999000300010 10502149

[4] Hacker M de A, Duppre NC, Nery JAC, Sales AM, Sarno EN. Characteristics of leprosy diagnosed through the surveillance of contacts: a comparison with index cases in Rio de Janeiro, 1987–2010. Mem Inst Oswaldo Cruz. Fundação Oswaldo Cruz; 2012;107: 49–54. doi: 10.1590/S0074-02762012000900009 10.1590/s0074-02762012000900009 23283453

[5] Beiguelman B. An Appraisal of Genetic Studies on Leprosy. Acta Genet Med Gemellol (Roma). Cambridge University Press; 1972;21: 21–52. doi: 10.1017/S1120962300011094

[6] Smith DG. The genetic hypothesis for susceptibility to lepromatous leprosy. Hum Genet. 1979;50: 163–77. Available: http://www.ncbi.nlm.nih.gov/pubmed/511131 511131 10.1007/BF00390238

[7] Moet FJ, Pahan D, Schuring RP, Oskam L, Richardus JH. Physical Distance, Genetic Relationship, Age, and Leprosy Classification Are Independent Risk Factors for Leprosy in Contacts of Patients with Leprosy. J Infect Dis. 2006;193: 346–353. doi: 10.1086/499278 16388481 10.1086/499278

[8] Moura MLN, Dupnik KM, Sampaio GAA, Nóbrega PFC, Jeronimo AK, do Nascimento-Filho JM, et al Active surveillance of Hansen’s Disease (leprosy): importance for case finding among extra-domiciliary contacts. PLoS Negl Trop Dis. 2013;7: e2093 doi: 10.1371/journal.pntd.0002093 23516645 10.1371/journal.pntd.0002093 PMC3597486

[9] World Health Organization. Global leprosy update, 2014: need for early case detection. Wkly Epidemiol Rec. 2015; No. 36, 2015, 90, 461–476. 26343055

[10] Manaus, Fundação Alfredo da Matta. Boletim Epidemiologico, 2016 [Internet]. Manaus; 2016. http://www.fuam.am.gov.br/wp-content/uploads/2014/05/Boletim_2016.pdf

[11] Richardus JH, Oskam L. Protecting people against leprosy: Chemoprophylaxis and immunoprophylaxis. Clin Dermatol. 2015;33: 19–25. doi: 10.1016/j.clindermatol.2014.07.009 25432807 10.1016/j.clindermatol.2014.07.009

[12] Sales AM, Ponce de Leon A, Düppre NC, Hacker MA, Nery JAC, Sarno EN, et al Leprosy among patient contacts: A multilevel study of risk factors. PLoS Negl Trop Dis. 2011;5. 10.1371/journal.pntd.0001013 PMC3057944 21423643

[13] Düppre NC, Camacho LAB, da Cunha SS, Struchiner CJ, Sales AM, Nery JAC, et al Effectiveness of BCG vaccination among leprosy contacts: a cohort study. Trans R Soc Trop Med Hyg. 2008;102: 631–638. doi: 10.1016/j.trstmh.2008.04.015 18514242 10.1016/j.trstmh.2008.04.015

[14] Brasil. Ministério da Saúde. Secretaria de Vigilância em Saúde. Diretrizes para vigilância, atenção e eliminacao da hanseníase como problema de saúde pública: manual técnico-operacional. Brasil, Saúde M da, Saúde S de V em, editors. Brasilia: Editora MS; 2016.

[15] Araújo MG, Lana FCF, Fonseca P de TS, Lanza FM. Leprosy incidence among children at Belo Horizonte city from 1992 to 1999: control implications. Rev méd Minas Gerais. 2016;14: 78–83.

[16] Ezenduka C, Post E, John S, Suraj A, Namadi A, Onwujekwe O. Cost-effectiveness analysis of three leprosy case detection methods in Northern Nigeria. PLoS Negl Trop Dis. 2012;6: e1818 doi: 10.1371/journal.pntd.0001818 23029580 10.1371/journal.pntd.0001818 PMC3447964

[17] Talhari S, Torrecila MA, Talhari AC. A study of leprosy and other skin diseases in school children in the state of Amazonas, Brazil. Lepr Rev. 1987;58: 233–7. 3669861 10.5935/0305-7518.19870025

[18] Brasil, Instituto Brasileiro de Geografia e Estatistica. Estimativas da população residente no Brasil e Unidades da Federação com data de referencia em 1° de julho de 2017 [Internet]. 2017 [cited 2 Nov 2017]. ftp://ftp.ibge.gov.br/Estimativas_de_Populacao/Estimativas_2017/estimativa_tcu_2017.pdf

[19] Instituto Brasileiro de Geografia e Estatistica. In: Cidades [Internet]. 2017 [cited 4 May 2017]. http://www.cidades.ibge.gov.br/v4/brasil/am/manaus/panorama

[20] Ridley DS, Jopling WH. A classification of leprosy for research purposes. Lepr Rev. 1962;33: 119–28. Available: http://www.ncbi.nlm.nih.gov/pubmed/14492126 14492126 10.5935/0305-7518.19620014

[21] Ridley DS. Histological classification and the immunological spectrum of leprosy. [Internet]. Bulletin of the World Health Organization. World Health Organization; 1974 http://www.ncbi.nlm.nih.gov/pubmed/4549496 PMC2366326 4549496

[22] Martinez AN, Talhari C, Moraes MO, Talhari S. PCR-based techniques for leprosy diagnosis: from the laboratory to the clinic. PLoS Negl Trop Dis. Public Library of Science; 2014;8: e2655 doi: 10.1371/journal.pntd.0002655 24722358 10.1371/journal.pntd.0002655 PMC3983108

[23] World Health Organization. WHO | WHO recommended MDT regimens [Internet]. WHO. World Health Organization; 2016 http://www.who.int/lep/mdt/regimens/en/#.WgtBWl-ax5U.mendeley

[24] Brasil, Ministério da Saúde, Secretaria de Vigilância em Saúde. Indicadores epidemiológicos e operacionais de hanseníase [Internet]. Brasilia; 2016. http://portalarquivos.saude.gov.br/images/pdf/2017/julho/10/Indicadores-epidemiol--gicos-e-operacionais-de-hansen--ase.Brasil,2001-.pdf

[25] Brasil, Instituto Brasileiro de Geografia e Estatistica. IBGE | Projeção da população [Internet]. 2013 [cited 7 Jan 2018]. https://ww2.ibge.gov.br/apps/populacao/projecao/

[26] Ministério da Saúde, DATASUS, RIPSA. Indicadores Demograficos—Taxa de Natalidade [Internet]. 2012 [cited 9 Jan 2018]. http://tabnet.datasus.gov.br/cgi/idb2012/a07a.htm

[27] Barreto JG, Bisanzio D, de Guimarães LS, Spencer JS, Vazquez-Prokopec GM, Kitron U, et al Spatial Analysis Spotlighting Early Childhood Leprosy Transmission in a Hyperendemic Municipality of the Brazilian Amazon Region. PLoS Negl Trop Dis. Public Library of Science; 2014;8. 10.1371/journal.pntd.0002665 PMC3916250 24516679

[28] Frade MAC, de Paula NA, Gomes CM, Vernal S, Bernardes Filho F, Lugão HB, et al Unexpectedly high leprosy seroprevalence detected using a random surveillance strategy in midwestern Brazil: A comparison of ELISA and a rapid diagnostic test. Johnson C, editor. PLoS Negl Trop Dis. 2017;11: e0005375 doi: 10.1371/journal.pntd.0005375 28231244 10.1371/journal.pntd.0005375 PMC5358972

[29] Wesley SR, Nair GT, Nair B. Leprosy among school children in Trivandrum city. Indian J Dermatol Venereol Leprol. Indian Journal of Dermatology, Venereology and Leprology (IJDVL); 1990;56: 288.

[30] Santos SD, Penna GO, Costa M da CN, Natividade MS, Teixeira MG. Leprosy in children and adolescents under 15 years old in an urban centre in Brazil. Mem Inst Oswaldo Cruz. Instituto Oswaldo Cruz; 2016;111: 359–64. doi: 10.1590/0074-02760160002 27223655 10.1590/0074-02760160002 PMC4909033

[31] Fine PE, Sterne JA, Pönnighaus JM, Bliss L, Saui J, Chihana A, et al Household and dwelling contact as risk factors for leprosy in northern Malawi. Am J Epidemiol. 1997;146: 91–102. 9215227 10.1093/oxfordjournals.aje.a009195

[32] Lana FCF, Amaral EP, Lanza FM, Lima PL, de Carvalho ACN, Diniz LG. Hanseníase em menores de 15 anos no Vale do Jequitinhonha, Minas Gerais, Brasil. Rev Bras Enferm. 2007;60: 696–700. doi: 10.1590/S0034-71672007000600014 18472544 10.1590/s0034-71672007000600014

[33] Ferreira IN, Alvarez RRA. Hanseníase em menores de quinze anos no município de Paracatu, MG (1994 a 2001). Rev Bras Epidemiol. 2005;8: 41–49. doi: 10.1590/S1415-790X2005000100006

[34] Franco MCA, Xavier MB, Franco ACA, Jucá Neto FOM, de Menezes BQ, Macedo GMM. Perfil de casos e fatores de risco para hanseníase, em menores de quinze anos, em município hiperendêmico da região norte do Brasil. Rev para med. 2014;28.

[35] Fernandes KB, Alves DM, de Mangueira J O. Fatores de risco para a transmissão da hanseníase. Rev Digit Buenos Aires. 2014;195: 12 Available: http://www.efdeportes.com

[36] Durães SMB, Guedes LS, da Cunha MD, Cavaliere FAM, de Oliveira MLWDR. Estudo de 20 focos familiares de hanseníase no município de Duque de Caxias, Rio de Janeiro. An Bras Dermatol. 2005;80: S295–S300.

[37] Madarasingha NP, Senaviratne JKK. A study of household contacts of children with leprosy. Ceylon Med J. 2011;56: 112–4. Available: http://www.ncbi.nlm.nih.gov/pubmed/22164748 22164748 10.4038/cmj.v56i3.3602

[38] Cunha SS, Alexander N, Barreto ML, Pereira ES, Dourado I, de Fátima Maroja M, et al BCG Revaccination Does Not Protect Against Leprosy in the Brazilian Amazon: A Cluster Randomised Trial. Daumerie D, editor. PLoS Negl Trop Dis. 2008;2: e167 doi: 10.1371/journal.pntd.0000167 18270542 10.1371/journal.pntd.0000167 PMC2238709

[39] de Carvalho FM, Rodrigues LS, Duppre NC, Alvim IMP, Ribeiro-Alves M, Pinheiro RO, et al Interruption of persistent exposure to leprosy combined or not with recent BCG vaccination enhances the response to Mycobacterium leprae specific antigens. Azman AS, editor. PLoS Negl Trop Dis. 2017;11: e0005560 doi: 10.1371/journal.pntd.0005560 28467415 10.1371/journal.pntd.0005560 PMC5432189

[40] World Health Organization. Global leprosy strategy 2016–2020: accelerating towards a leprosy-free world. [Internet]. New Delhi; 2016. 978-92-9022-509-6

[41] Steinmann P, Reed SG, Mirza F, Hollingsworth TD, Richardus JH. Innovative tools and approaches to end the transmission of Mycobacterium leprae. Lancet Infect Dis. 2017;17: e298–e305. doi: 10.1016/S1473-3099(17)30314-6 28693856 10.1016/S1473-3099(17)30314-6

